# MiR-208a stimulates the cocktail of SOX2 and β-catenin to inhibit the let-7 induction of self-renewal repression of breast cancer stem cells and formed miR208a/let-7 feedback loop via LIN28 and DICER1

**DOI:** 10.18632/oncotarget.5079

**Published:** 2015-10-08

**Authors:** Xin Sun, Shiwen Jiang, Jian Liu, Huangzhen Wang, Yiwen Zhang, Shou-Ching Tang, Jichang Wang, Ning Du, Chongwen Xu, Chenguang Wang, Sida Qin, Jia Zhang, Dapeng Liu, Yunfeng Zhang, Xiaojun Li, Jiansheng Wang, Jun Dong, Xin Wang, Shaohua Xu, Zhen Tao, Fei Xu, Jie Zhou, Tao Wang, Hong Ren

**Affiliations:** ^1^ Department of Thoracic Surgery and Oncology, the First Affiliated Hospital of Xi'an Jiaotong University, Xi'an, Shaanxi Province, 710061, China; ^2^ Department of Obstetrics and Gynecology, the Second Affiliated Hospital of Wenzhou Medical University, Wenzhou, Zhejiang Province, 325027, China; ^3^ Breast Cancer Program and Interdisciplinary Translational Research Team, Georgia Regents University Cancer Center, Augusta, Georgia, 30912, United States; ^4^ Tianjin Medical University Cancer Institute and Hospital, Tianjin, 300060, China; ^5^ Neurosurgery Department of the First Affiliated Hospital of Xi'an Jiaotong University, Xi'an, Shaanxi Province, 710061, China; ^6^ Institute of Radiation Medicine, the Chinese Academy of Medical Sciences, Nankai District, Tianjing 300192, China; ^7^ Department of Orthopaedics, the Second Affiliated Hospital of Xi'an Jiaotong University, Xi'an, Shaanxi Province, 710061, China; ^8^ Department of Gastroenterology, the First Affiliated Hospital of Xi'an Jiaotong University, Xi'an, Shaanxi Province, 710061, China; ^9^ Department of Gynecology, Shanghai First Maternity and Infant Hospital, Tongji University School of Medicine, Shanghai, 201204, China; ^10^ Department of Radiation Oncology, Tianjin Cancer Institute and Hospital Affiliated to Tianjin Medical University, Tianjin, 300060, China; ^11^ Department of Radiation Oncology, Shanghai Cancer Center, Fudan University, Shanghai, 200032, China; ^12^ Department of Breast Oncology, Affiliated Cancer Hospital of Guangzhou Medical University, Guangzhou, Guangdong Province, 510182, China; ^13^ Department of Urology, Tongji Hospital, Tongji Medical College, Huazhong University of Science and Technology, Wuhan, Hubei Province, 430030, China

**Keywords:** cancer stem cells, feedback loop, breast tumor, miRNA-208a, let-7a

## Abstract

MiR-208a stimulates cardiomyocyte hypertrophy, fibrosis and β-MHC (β-myosin heavy chain) expression, being involved in cardiovascular diseases. Although miR-208a is known to play a role in cardiovascular diseases, its role in cancer and cancer stem cells (CSCs) remains uncertain. We identified an inverse relationship between miR-208a and let-7a in breast cancer specimens, and found that SOX2, β-catenin and LIN28 are highly expressed in patients with advanced breast cancer opposed to lesser grades. Further, we isolated ALDH1+ CSCs from ZR75–1 and MDA-MB-231 (MM-231) breast cancer cell lines to test the role of miR-208a in breast CSCs (BrCSCs). Our studies showed that overexpression of miR-208a in these cells strongly promoted the proportion of ALDH1+ BrCSCs and continuously stimulated the self-renewal ability of BrCSCs. By using siRNAs of SOX2 and/or β-catenin, we found that miR-208a increased LIN28 through stimulation of both SOX2 and β-catenin. The knockdown of either SOX2 or β-catenin only partially attenuated the functions of miR-208a. Let-7a expression was strongly inhibited in miR-208a overexpressed cancer cells, which was achieved by miR-208a induction of LIN28, and the restoration of let-7a significantly inhibited the miR-208a induction of the number of ALDH1+ cells, inhibiting the propagations of BrCSCs. In let-7a overexpressed ZR75–1 and MM-231 cells, DICER1 activity was significantly inhibited with decreased miR-208a. Let-7a failed to decrease miR-208a expression in ZR75–1 and MM-231 cells with DICER1 knockdown. Our research revealed the mechanisms through which miR-208a functioned in breast cancer and BrCSCs, and identified the miR-208a-SOX2/β-catenin-LIN28-let-7a-DICER1 regulatory feedback loop in regulations of stem cells renewal.

## INTRODUCTION

MiR-208a is thought to be mainly expressed in the heart, as a response to stress and hypothyroidism [1, 2]. MiR-208a is encoded by an intron of the alpha-myosin heavy chain (α-MHC) [3], stimulating cardiomyocyte hypertrophy, fibrosis and β-MHC expression [4]. However, roles of miR-208a in malignancies are still unclear. Recent research focused on human esophageal squamous cell carcinoma and pancreatic cancers revealed oncogenic roles of miR-208a in human tumors; however, the studies did not address the mechanisms through which miR-208a worked in cancer and cancer stem cells (CSCs) [5, 6]. In this study, we focused on the role of miR-208a in promoting breast cancer, especially in terms of its effect on breast cancer stem cells (BrCSCs) self-renewal, and studied the possible mechanisms related to oncogenic miR-208a. A negative feedback loop of miR-208a and its downstream genes was identified in this study, which all play critical roles in the regulation of stem cells renewal.

## RESULTS

### The correlation between SOX2/β-catenin and LIN28 in clinical breast tumors

**Figure 1:**
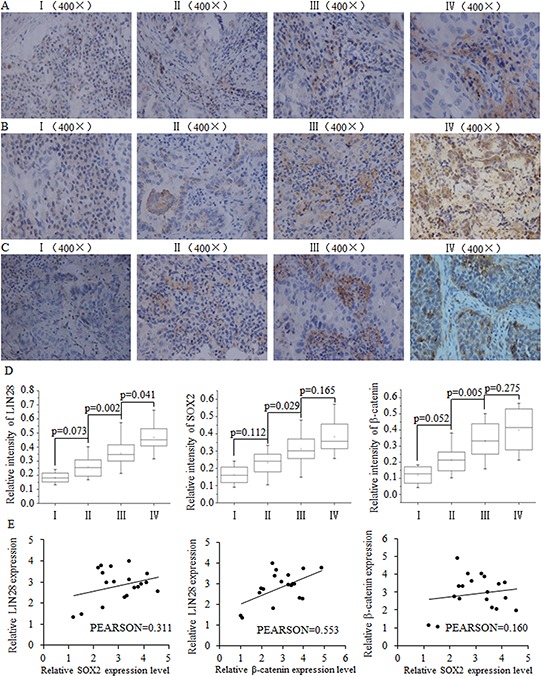
The positive correlation between SOX2/β-catenin and LIN28 and their clinical value in evaluating prognosis of patients with breast cancer Representative examples of immunohistochemical staining for LIN-28 **A.** SOX2 **B.** and β-catenin **C.** in each of the clinical stages of breast cancers as indicated. Stage I had the lowest expression of LIN28, SOX2, and β-catenin. **D.** Quantification of LIN-28, SOX2, and β-catenin relative immunostaining intensity for each clinical stage of breast cancers. Data is shown as mean ± SEM. **E.** Relative expression levels of SOX2 and LIN28, β-catenin and LIN28, and SOX2 and β-catenin, with correlation coefficients shown in figure.

We analyzed the expression levels of LIN28, SOX2 and β-catenin in breast cancer samples of different stages from 35 breast cancer patients. The immunohistochemistry results indicated a correlation between the expression of LIN28, SOX2 and β-catenin protein and later-stage breast tumors (Fig. 1A–1D). At the mRNA level in breast tumor tissues, a positive correlation was identified between Lin28 and SOX2 as well as between LIN28 and β-catenin, but not between β-catenin and SOX2 (Fig. 1E)

### Inverse correlation between let-7a miRNA and miR-208a

**Figure 2:**
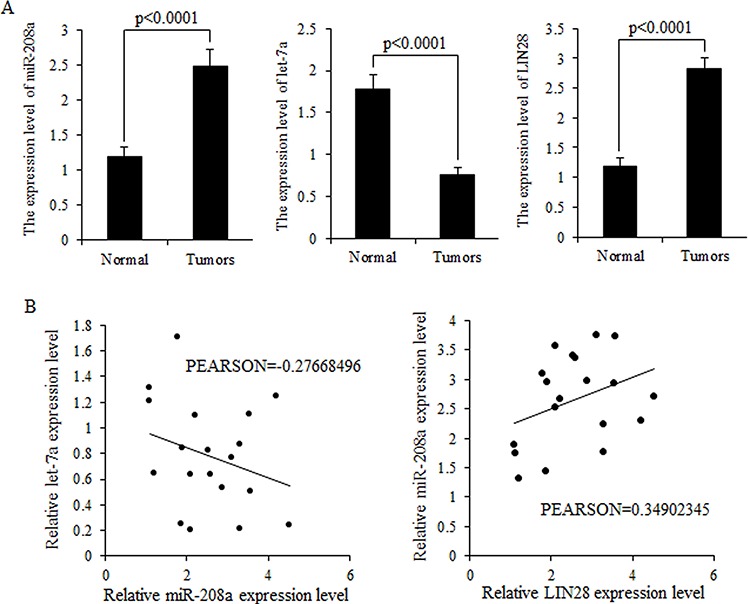
Expression levels of miR-208a and LIN28 mRNA are inversely correlated with that of let-7a in tumors **A.** Relative expression levels of let-7a miRNA, miR-208a and LIN28 mRNA in breast cancer specimens, compared to normal tissues. LIN28 abundance was normalized to 18S rRNA. Let-7a and miR-208a levels were measured by TaqMan stem-loop qRT-PCR, and U6 was set as internal control. Data is shown as mean ± SEM. **B.** Let-7a and miR-208a was inversely correlated in human breast cancer; however, miR-208a and LIN28 are positively related to each other.

MiR-208a and LIN28 mRNA were highly expressed in breast cancer tissues, but let-7 was strongly inhibited in breast malignancies (Fig. 2A). Higher expression levels of miR-208a correlated with lower expression levels of let-7a mRNA but higher LIN28 mRNA expression (Fig. 2B).

### MiR-208a promotes the self-renewal ability of breast cancer stem cells

**Figure 3:**
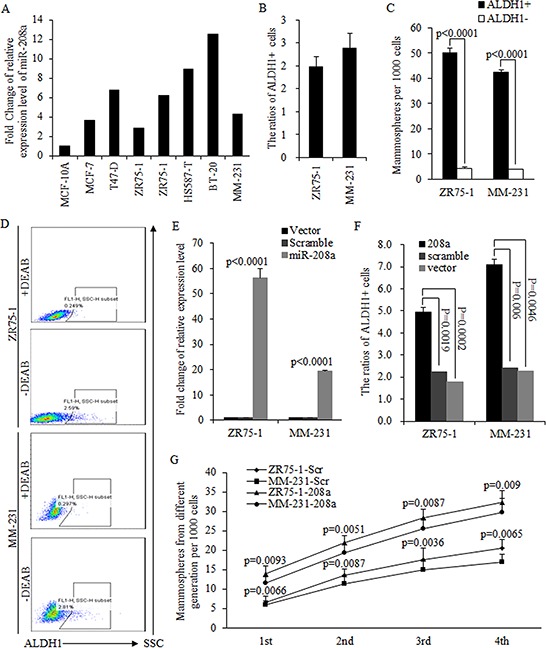
MiR-208a promotes the self-renewal ability of breast cancer stem cells **A.** MiR-208a was universally upregulated in multiple breast cancer cell lines, compared to normal MCF-10A cell line, and miR-208a was lower expressed in ZR75–1 and MM-231 cells relatively. **B.** The proportion of ALDH1+ cells in ZR75–1 and MM-231 cells. **C.** ALDH1+ cells are capable of forming more mammospheres compared to ALDH1- cells. **D.** Images of FACS sorting analysis of ALDH1+ breast cancer stem cells in ZR75–1 and MM-231 cells. Overexpressed miR-208a in ZR75–1 and MM-231 cells **E.** significantly increased the ratios of ALDH1+ breast cancer stem cells **F. G.** MiR-208a continuously stimulated the self-renewal ability of cancer stem cell in four generations of mammospheres.

To study the function of miR-208a in breast tumors, we first detected the endogenous level of miR-208a in multiple breast cancer cell lines, and found lower miR-208a expression in estrogen receptor positive ZR75–1 and triple negative MDA-MB-231 (MM-231) cells, compared to other breast cancer cell lines (Fig. 3A). To identify the role of miR-208a in BrCSCs, we used ALDH1-based FACS sorting, where ALDH1+ cells are more capable of self-renewal compared to ALDH1- cells (Fig. 3B–3D). Overexpression of miR-208a in ZR75–1 and MM-231 (Fig. 3E) strongly correlates with the high proportion of ALDH1+ BrCSCs (Fig. 3F). Further, we studied the role of miR-208a on continuously cultured mammospheres as a model, for identifying the capacity of self-renewal; results indicated that miR-208a stimulated the self-renewal ability of mammospheres in up to four generations (Fig. 3G).

### MiR-208a's stimulations of LIN28 functions through promoting SOX2 and β-catenin in BrCSCs

**Figure 4:**
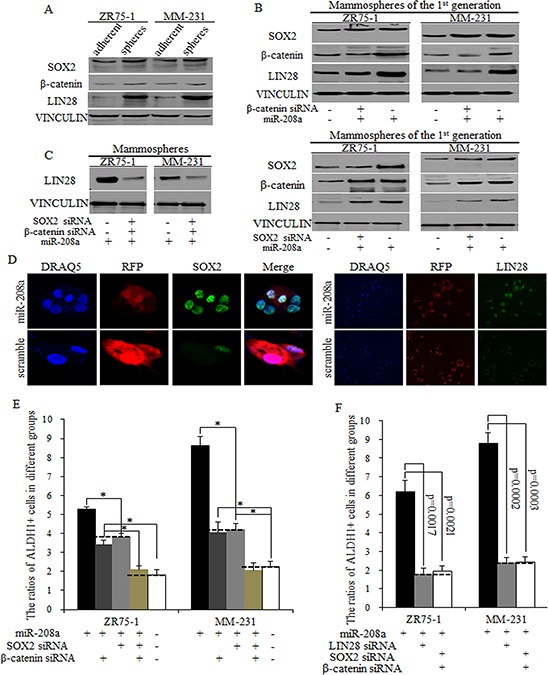
MiR-208a promotes LIN28 via regulations of SOX2 and β-catenin **A.** SOX2, β-catenin and LIN28 are upregulated in mammospheres from ZR75–1 and MM-231 cells, compared to adherent cells. **B.** miR-208a stimulates LIN28 expression via SOX2 and β-catenin partially, and the inhibition of SOX2 or β-catenin could only partially functions of miR-208a in induction of LIN28. **C.** siRNAs of SOX2 and β-catenin together effectively inhibited LIN28 expression in miR-208a overexpressed ZR75–1 and MM-231 cells. **D.** SOX2 and LIN28 increased in miR-208a overexpressed breast cancer stem cells. **E.** The inhibition of SOX2 or β-catenin decreased the ratio of ALDH1+ stem cells; this inhibition was significantly greater when both SOX2 and β-catenin siRNA was used, reducing the ratio of ALDH1+ cells to control levels. **F.** No significant differences of ratios of ALDH1+ cells were detected in groups of LIN28 inhibition and SOX2/β-catenin inhibition.

The downstream genes of miR-208a were identified on http://www.TargetScan.com and pasted to the David data base (http://david.abcc.ncifcrf.gov/summary.jsp). We found that miR-208a may stimulate Wnt signaling (Fig. S1A) and the EMT pathway (Fig. S1B), with the Wnt signaling pathway being highly predicated (Fig. S1A). We first checked the increased expression levels of SOX2, β-catenin, and LIN28 in spheres from ZR75–1 and MM-231 cells (Fig. 4A). To further identify the mechanism of miR-208a in stimulating BrCSCs, we used three pairs of siRNAs of SOX2 and β-catenin. Results showed that miR-208a increased the ratio of ALDH1+ stem cells through increasing SOX2 and β-catenin expression in mammospheres, and then increased LIN28 level synergistically (Fig. 4B–4D). Inhibition of SOX2 or β-catenin effectively weakened miR-208a function, diminished the self-renewal capacity, but not to control levels (Fig. 4E). In contrast, the combined use of siRNAs of SOX2 and β-catenin almost entirely blocked miR-208a function, and no obvious differences were observed between miR-208a and control group, indicating the combination of SOX2 and β-catenin inhibition were enough and essential to reverse miR-208 function (Fig. 4E).

LIN28 is a strong transcription factor that promotes self-renewal, multipotent and differentiation, and capable of stimulating the amplification of stem cells group. SOX2 and β-catenin have been shown to stimulate LIN28, exerting oncogenic effects on stem cell renewal. We found that miR-208a increased LIN28 level through stimulation of both SOX2 and β-catenin (Fig. 4B), and the inhibition of either SOX2 or β-catenin weakened miR-208a function to a certain extent (Fig. 4E). The combined inhibition of SOX2 and β-catenin showed the same effects as the inhibition of LIN28 did, which suggests that miR-208a functioned through SOX2/β-catenin-LIN28 signaling pathway (Fig. 4F).

### MiR-208a represses mature let-7a expression via stimulation of LIN28

**Figure 5:**
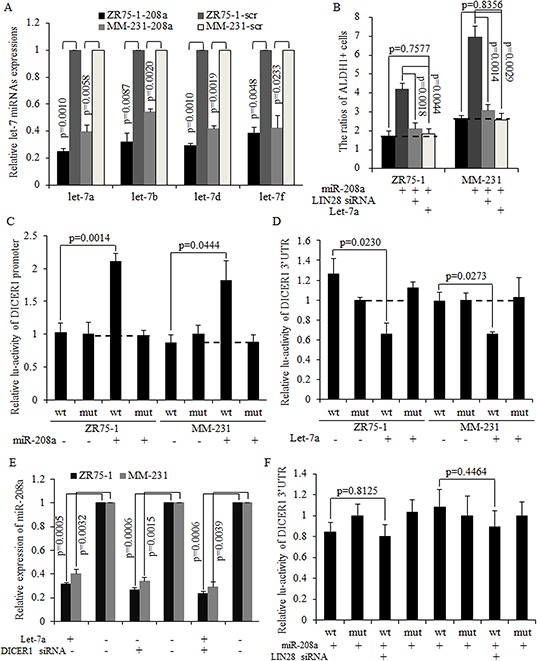
miR-208a functioned through decreasing let-7 expression via LIN28 and formed miR-208a/LIN28/let-7a feedback loop **A.** Let-7a expression is significantly decreased in miR-208a-overexpressing breast cancer cells. **B.** The LIN28 inhibition and let-7a overexpression both decreased the number of ALDH1+ stem cells. **C.** MiR-208a increased the activity of the DICER1 promoter. **D.** Let-7a directly targeted and degraded DICER1 mRNA by binding to the 3′UTR. **E.** Let-7a decreased miR-208a expression level through inhibiting DICER1. **F.** The inhibition of LIN28 decreased the activity of DICER1 3′UTR via increasing let-7 expression in miR-208a-overexpressing breast cancer cells.

LIN28 is a strong inhibitor of the let-7 family of miRNAs [22]. Mature let-7a/b/d/f were found to have decreased expression in miR-208a overexpressed cancer cells (Fig. 5A), with let-7a expression being the most strongly inhibited one. The restoration of mature let-7a significantly inhibited the miR-208a induction of the number of BrCSCs, and the same results were observed when LIN28 was silenced using siRNA (Fig. 5B).

### MiR-208a and let-7a form a negative feedback loop via LIN28 and DICER1

It has been reported that LIN28 repressed let-7a maturation, forming a regulatory loop with let-7a directly [22–24]. We hypothesized that decreased let-7a was required for oncogenic roles of miR-208a in breast cancer stem cells. Mature miRNAs are processed by DICER1 and released to the cytoplasm as a functional unit of the RNA-induced silencing complex (RISC) [25]. As let-7could target at DICER1 as downstream mRNA [26], we then hypothesized that let-7a may inhibit miR-208a through decreasing DICER1. The overexpression of miR-208a effectively promotes the DICER promoter activity (Fig. 5C). In let-7a overexpressed ZR75–1 and MM-231 cells, expressions of DICER1 mRNA and miR-208a are significantly decreased (Fig. 5D–5E). Let-7a also abolished the oncogenic functions of miR-208a in stimulating the self-renewal ability of stem cells (Fig. S2A–S2B). Let-7a overexpression and DICER1 silencing resulted in miR-208a expression decreasing to a similar level as when either let-7a is overexpressed or DICER1 is silenced (Fig. 5E). However, let-7a failed to decrease miR-208a expression in DICER1 knockdown ZR75–1 and MM-231 cells (Fig. 5E). The inhibition of LIN28 abolished miR-208a induction of DICER1 mRNA (Fig. 5F), and exhibited the same effects on stem cell number as let-7a did (Fig. S2C). These results indicate that miR-208a increased DICER1 expression through the LIN28-let-7a pathway, and that LIN28 functioned through inhibiting let-7a in mammospheres (Fig. S3).

## DISCUSSION

The role of miRNAs in cancer has been widely explored in the past several years, and many networks involving miRNAs and genes have been established, expanding the understanding of cellular regulation in tumor biology and, adding to the known oncogenic or suppressive signaling pathways [27]. Thousands of miRNAs have been identified since they were first discovered, and miRNAs take part in many physiological functions and pathological reactions. MiR-208a was previously known only with respect to cardiovascular disease, although the oncogenic role of miR-208a was recently tentatively explored in hepatocellular carcinoma and in esophageal tumors, the results were circumscribed and the role of miR-208a in breast cancer or in cancer stem cells is not known. LIN28 is a translational enhancer and transcription factor that forms a trimeric complex with OCT4 on DNA, controlling the expression of a number of genes by increasing the efficiency of protein synthesis [28, 29]. LIN28 suppresses the biogenesis of let-7 miRNAs by specifically recognizing and binding to the motif in the terminal loop of pre-let-7 [30, 31], and then recruiting the ZCCHC11/TUT4 uridylyltransferase, leading to the terminal uridylation of pre-let-7 [32].

In this study, we hypothesized that miR-208a may act as oncogene in breast tumor through interacting with LIN28 and let-7a. We then detected the endogenous miR-208a expression level in multiple breast cancer cell lines, and found decreased miR-208a expression in ZR75–1 (ER+) and MDA-MB-231 (triple negative) cells. We then used ALDH1 based FACS sorting and mammosphere forming assays to enrich for BrCSCs. MiR-208a significantly increased the number of BrCSCs, and stimulated their self-renewal ability in continuously cultured mammospheres, strongly suggesting the role for miR-208a in tumorigenesis and malignancy. Through bioinformatic analysis and target gene predication, we hypothesized that miR-208a may stimulate SOX2 and β-catenin to influence the self-renewal ability of stem cells. Although inhibiting SOX2 or β-catenin alone was not sufficient to account for miR-208a's induction of stemness, inhibiting both SOX2 and β-catenin was.

LIN28 is not predicated as a downstream gene of miR-208a, but could interact with SOX2 and β-catenin to play a critical role in promoting pluripotency [23, 33, 34]. Inhibiting SOX2 and β-catenin significantly decreased the LIN28 expression. Increased LIN28 attributed to miR-208a functions through decreasing the expression of mature let-7 miRNAs. The let-7 family is a strong promoter of cell differentiation, governing the normal status of an organism's development, and acting as suppressive miRNAs in tumors [35]. In our studies, we found that miR-208a functioned through promoting LIN-28 via both SOX2 and β-catenin, which eventually inhibited the maturation of let-7a. We also determined that miR-208a and let-7a formed a regulatory loop through LIN28 and DICER1, both of which are crucial regulators in biogenesis in the process of miRNA maturation. Our research revealed the mechanism through which miR-208a may function in malignancy and in CSCs, identifying the miR-208a-SOX2/β-catenin-LIN28-let-7a regulatory feedback loop. Cocktail style of gene regulation is common in pathophysiology performance, and miRNAs may function through interacting with multiple genes, not only by targeting genes that are direct downstream ones, and also indicated that the regulatory effects of miRNA on miRNAs be one of the crucial mechanisms that miRNA may function through.

## MATERIALS AND METHODS

### Cell culture, transfection, lentiviral infection, luciferase assay and Fluorescence-activated cell sorting analysis

Breast cancer cell lines (MCF-7, T47-D, ZR75–1, HS587-T, BT-20 and MDA-MB-231) and a normal breast cell line (MCF-10A) were purchased from ATCC and kept at the Central Laboratory affiliated to Xi'an Jiaotong University School of Medicine and Center for Translational Medicine of the First Affiliated Hospital of Xi'an Jiaotong University. Breast cancer cells were cultured in DMEM 1x (Cellgro, CORNING, USA) medium, supplemented with 10%~15% Fetal Bovine Serum (FBS, Gibco, Life Technologies, USA), 1% penicillin and streptomycin (Cellgro, CORNING, USA). ALDH1+ CSCs and mammospheres were cultured in DMEM/Ham's F-12 medium and supplemented with 10 μg/ml epidermal growth factor (EGF, Invitrogen, USA), 10 ng/ml human basic fibroblast growth factor (hbFGF, Invitrogen, USA), 10 ng/ml hydrocortisone (Invitrogen, USA) and 4 μg/ml insulin (Invitrogen, USA) [7]. MCF-10A was cultured in DMEM/Ham's F-12 medium supplemented with 5% FBS (Gibco, Life Technologies, USA), 20 ng/ml EGF, 0.5 mg/ml Hydrocortisone, 100 ng/ml Cholera Toxin, 10 μg/ml insulin [7]. All cells were cultured in 5% CO_2_ at 37°C. Oligonucleotides encoding mature miRNAs (miR-208a, let-7a, scramble control) were synthesized and cloned into the pGLVU6/RFP vector (GenePharma Inc., Shanghai, China). Transfections of siRNAs and luc-plasmids were performed using Lipofectamine 2000 (Invitrogen, USA), and cells of different groups were seeded at 50% confluence in a 24-well plate prior to transient transfection [8]. The activated ALDEFLUOR™ reagent and DEAM (Stem Cell Technologies, USA) were used to isolate ALDH1+ stem cells from breast cancer cell lines [9–11], and ratios of ALDH1+ CSCs in different groups were analyzed.

### Quantitative real-time PCR and western blot

Clinical specimens were collected from patients of the First Affiliated Hospital of Xi'an Jiaotong University, and informed consent of all patients was obtained, allowing the use of tumor tissues and adjacent normal breast tissue for diagnosis and medical studies. Total RNA was extracted from clinical specimens or cultured cells using Trizol Reagent (Life Technology, USA) [12, 13]. Real-time quantitative reverse transcription polymerase chain reaction (qRT-PCR) was performed to detect miRNA expression levels, and SYBR green was used to detect PCR products; reactions were performed in triplicate using a 25 μl reaction volume [12, 14]. Expression levels were calculated and presented following the 2^−ΔΔCt^ method (ΔΔCt = ΔCt (positive)-ΔCt (control)) [15, 16]. Total protein was extracted and transferred as previously described [13, 17]. In brief, the membranes were incubated overnight with specific primary antibodies to SOX2 (1:1000, ab92494, Abcam, USA), LIN28 (1:1000, AP1485A, ABGENT, CA, USA), β-catenin (1:2000, ab78483, Abcam, USA), and Vinculin (1:5000, #4650, Cell Signaling, USA). The secondary antibodies were conjugated with HRP (1:5000, Santa Cruz, USA) for 1 h.

### Immunofluorescence and immunohistochemical staining

The cells were fixed in 10% formaldehyde for 15 min and then incubated in 1% BSA, 10% normal goat serum and 0.3 M glycine in 0.1% PBST for 1 h to permeabilize the cells and block non-specific protein-protein interactions. Immunofluorescence staining used SOX2 (1:200, ab92494, Abcam, USA) and LIN28 (1:100, AP1485A, ABGENT, USA); the slide was incubated with a secondary antibody for 30 min with Alexa Fluor^®^488 goat anti-rabbit IgG (H+L) used at a 1/1000 dilution for 1 h at room temperature. Cells were counterstained with DRAQ5 (1:10000; 4084; Cell Signaling, China) to show nuclei. For immunohistochemical staining, paraffin tissue sections were deparaffinized with xylol and then rehydrated in an alcohol gradient and rinsed in deionized water. We chose the high pressure method for antigen retrieval, and then cooled the slides down to room temperature. A 3% hydrogen peroxide solution was used to block the endogenous peroxidase, and then added a resistance after blocking by goat serum. The slides were refrigerated overnight at 4°C, and the slide was incubated with secondary antibody for 60 min the next day [13, 17].

### Sphere-formation assays

After trypsin enzyme digestion, cells were plated in ultra-low attachment dishes (Corning Incorporated, Lowell, MA, USA) without fetal bovine serum, to test their ability to form primary mammospheres. On the eighth day, the sphere number was counted. The mammosphere forming efficiency (MFE) was calculated as the ratio of obtained spheres verses number of plated cells (mean ± SEM) [18–21]. Obtained mammospheres were disaggregated into single cells using Accutase (StemPro, GIBCO, USA), and then re-suspended to acquire the next generation of mammospheres. Up to four generations of mammospheres were obtained to test the self-renewal ability of BrCSCs.

### Statistical analysis

All statistical analyses were performed using EXCEL 2010 (Microsoft, USA). All *in vitro* experiments were performed in triplicate, and all data were represented as mean ± SEM. Statistical analyses were conducted using Student's *t* test and Pearson Correlation Coefficient. The statistical significance of each value was set at *p* < 0.05.

## SUPPLEMENTARY MATERIALS FIGURES



## References

[1] Van Rooij E, Sutherland LB, Qi X, Richardson JA, Hill J, Olson EN (2007). Control of Stress-Dependent Cardiac Growth and Gene Expression by a MicroRNA. Science.

[2] Thorsen SB, Obad S, Jensen NF, Stenvang J, Kauppinen S (2012). The therapeutic potential of microRNAs in cancer. Cancer J.

[3] Wang GK, Zhu JQ, Zhang JT, Li Q, Li Y, He J, Qin YW, Jing Q (2010). Circulating microRNA: a novel potential biomarker for early diagnosis of acute myocardial infarction in humans. Eur Heart J.

[4] Grueter CE, van Rooij E, Johnson BA, DeLeon SM, Sutherland LB, Qi X, Gautron L, Elmquist JK, Bassel-Duby R, Olson EN (2012). A cardiac microRNA governs systemic energy homeostasis by regulation of MED13. Cell.

[5] Liu A, Shao C, Jin G, Liu R, Hao J, Song B, Ouyang L, Hu X (2014). miR-208-induced epithelial to mesenchymal transition of pancreatic cancer cells promotes cell metastasis and invasion. Cell Biochem Biophys.

[6] Li H, Zheng D, Zhang B, Liu L, Ou J, Chen W, Xiong S, Gu Y, Yang J (2014). Mir-208 promotes cell proliferation by repressing SOX6 expression in human esophageal squamous cell carcinoma. J Transl Med.

[7] Delort L, Perrier S, Dubois V, Billard H, Mracek T, Bing C, Vasson MP, Caldefie-Chezet F (2013). Zinc-alpha2-glycoprotein: a proliferative factor for breast cancer? *In vitro* study and molecular mechanisms. Oncol Rep.

[8] Sun X, Tang S-C, Xu C, Wang C, Qin S, Du N, Liu J, Zhang Y, Li X, Luo G, Zhou J, Xu F, Ren H (2015). DICER1 regulated let-7 expression levels in p53-induced cancer repression requires cyclin D1. Journal of Cellular and Molecular Medicine.

[9] Sun Y, Wang Y, Fan C, Gao P, Wang X, Wei G, Wei J (2014). Estrogen promotes stemness and invasiveness of ER-positive breast cancer cells through Gli1 activation. Mol Cancer.

[10] Ginestier C, Hur MH, Charafe-Jauffret E, Monville F, Dutcher J, Brown M, Jacquemier J, Viens P, Kleer CG, Liu S, Schott A, Hayes D, Birnbaum D, Wicha MS, Dontu G (2007). ALDH1 is a marker of normal and malignant human mammary stem cells and a predictor of poor clinical outcome. Cell stem cell.

[11] Liu SY, Zheng PS (2013). High aldehyde dehydrogenase activity identifies cancer stem cells in human cervical cancer. Oncotarget.

[12] Sun X, Qin S, Fan C, Xu C, Du N, Ren H (2013). Let-7: a regulator of the ERalpha signaling pathway in human breast tumors and breast cancer stem cells. Oncol Rep.

[13] Wang T, Liu Z, Guo S, Wu L, Li M, Yang J, Chen R, Xu H, Cai S, Chen H, Li W, Wang L, Hu Z, Zhuang Q, Xu S, Wang L (2014). The tumor suppressive role of CAMK2N1 in castration-resistant prostate cancer.

[14] Yu Z, Wang L, Wang C, Ju X, Wang M, Chen K, Loro E, Li Z, Zhang Y, Wu K, Casimiro MC, Gormley M, Ertel A, Fortina P, Chen Y, Tozeren A (2013). Cyclin D1 induction of Dicer governs microRNA processing and expression in breast cancer. Nat Commun.

[15] Liu H, Liang Y, Li Y, Wang J, Wu H, Wang Y, Tang SC, Chen J, Zhou Q (2010). Gene silencing of BAG-1 modulates apoptotic genes and sensitizes lung cancer cell lines to cisplatin-induced apoptosis. Cancer Biol Ther.

[16] He J, Wang M, Jiang Y, Chen Q, Xu S, Xu Q, Jiang BH, Liu LZ (2014). Chronic arsenic exposure and angiogenesis in human bronchial epithelial cells via the ROS/miR-199a-5p/HIF-1alpha/COX-2 pathway. Environ Health Perspect.

[17] Wang T, Guo S, Liu Z, Wu L, Li M, Yang J, Chen R, Liu X, Xu H, Cai S, Chen H, Li W, Xu S, Wang L, Hu Z, Zhuang Q (2014). CAMK2N1 inhibits prostate cancer progression through androgen receptor-dependent signaling.

[18] Cicalese A, Bonizzi G, Pasi CE, Faretta M, Ronzoni S, Giulini B, Brisken C, Minucci S, Di Fiore PP, Pelicci PG (2009). The tumor suppressor p53 regulates polarity of self-renewing divisions in mammary stem cells. Cell.

[19] Wu K, Jiao X, Li Z, Katiyar S, Casimiro MC, Yang W, Zhang Q, Willmarth NE, Chepelev I, Crosariol M, Wei Z, Hu J, Zhao K, Pestell RG (2011). Cell fate determination factor Dachshund reprograms breast cancer stem cell function. J Biol Chem.

[20] Liu M, Casimiro MC, Wang C, Shirley LA, Jiao X, Katiyar S, Ju X, Li Z, Yu Z, Zhou J, Johnson M, Fortina P, Hyslop T, Windle JJ, Pestell RG (2009). p21CIP1 attenuates Ras- and c-Myc-dependent breast tumor epithelial mesenchymal transition and cancer stem cell-like gene expression *in vivo*. Proceedings of the National Academy of Sciences.

[21] Lamb R, Harrison H, Smith DL, Townsend PA, Jackson T, Ozsvari B, Martinez-Outschoorn UE, Pestell RG, Howell A, Lisanti MP, Sotgia F (2015). Targeting tumor-initiating cells: eliminating anabolic cancer stem cells with inhibitors of protein synthesis or by mimicking caloric restriction. Oncotarget.

[22] Thornton JE, Gregory RI (2012). How does Lin28 let-7 control development and disease?. Trends in Cell Biology.

[23] Cai WY, Wei TZ, Luo QC, Wu QW, Liu QF, Yang M, Ye GD, Wu JF, Chen YY, Sun GB, Liu YJ, Zhao WX, Zhang ZM, Li BA (2013). The Wnt-beta-catenin pathway represses let-7 microRNA expression through transactivation of Lin28 to augment breast cancer stem cell expansion. J Cell Sci.

[24] Piskounova E, Polytarchou C, Thornton James E, LaPierre Robert J, Pothoulakis C, Hagan John P, Iliopoulos D, Gregory Richard I (2011). Lin28A and Lin28B Inhibit let-7 MicroRNA Biogenesis by Distinct Mechanisms. Cell.

[25] Redfern AD, Colley SM, Beveridge DJ, Ikeda N, Epis MR, Li X, Foulds CE, Stuart LM, Barker A, Russell VJ, Ramsay K, Kobelke SJ, Hatchell EC, Payne C, Giles KM, Messineo A (2013). RNA-induced silencing complex (RISC) Proteins PACT, TRBP, and Dicer are SRA binding nuclear receptor coregulators. Proc Natl Acad Sci U S A.

[26] Tokumaru S, Suzuki M, Yamada H, Nagino M, Takahashi T (2008). let-7 regulates Dicer expression and constitutes a negative feedback loop. Carcinogenesis.

[27] Sun X, Jiao X, Pestell TG, Fan C, Qin S, Mirabelli E, Ren H, Pestell RG (2014). MicroRNAs and cancer stem cells: the sword and the shield. Oncogene.

[28] Polesskaya A, Cuvellier S, Naguibneva I, Duquet A, Moss EG, Harel-Bellan A (2007). Lin-28 binds IGF-2 mRNA and participates in skeletal myogenesis by increasing translation efficiency. Genes & Development.

[29] Tan SM, Altschuler G, Zhao TY, Ang HS, Yang H, Lim B, Vardy L, Hide W, Thomson AM, Lareu RR (2014). Divergent LIN28-mRNA associations result in translational suppression upon the initiation of differentiation. Nucleic Acids Research.

[30] Mayr F, Heinemann U (2013). Mechanisms of Lin28-Mediated miRNA and mRNA Regulation—A Structural and Functional Perspective. International Journal of Molecular Sciences.

[31] Loughlin FE, Gebert LFR, Towbin H, Brunschweiger A, Hall J, Allain FHT (2012). Structural basis of pre-let-7 miRNA recognition by the zinc knuckles of pluripotency factor Lin28. Nat Struct Mol Biol.

[32] Thornton JE, Chang H-M, Piskounova E, Gregory RI (2012). Lin28-mediated control of let-7 microRNA expression by alternative TUTases Zcchc11 (TUT4) and Zcchc6 (TUT7). RNA.

[33] Cimadamore F, Amador-Arjona A, Chen C, Huang C-T, Terskikh AV (2013). SOX2-LIN28/let-7 pathway regulates proliferation and neurogenesis in neural precursors. Proceedings of the National Academy of Sciences.

[34] Gillis AJ, Stoop H, Biermann K, van Gurp RJ, Swartzman E, Cribbes S, Ferlinz A, Shannon M, Oosterhuis JW, Looijenga LH (2011). Expression and interdependencies of pluripotency factors LIN28, OCT3/4, NANOG and SOX2 in human testicular germ cells and tumours of the testis. Int J Androl.

[35] Xu C, Sun X, Qin S, Zheng Z, Xu S, Luo G, Liu P, Du N, Zhang Y, Liu D, Wang H, Chen K, Ren H (2015). Let-7a regulates mammosphere formation capacity through Ras/NF-kappaB and Ras/MAPK/ERK pathway in breast cancer stem cells. Cell Cycle.

