# Supplementary material for: MiR-208a stimulates the cocktail of SOX2 and β-catenin to inhibit the let-7 induction of self-renewal repression of breast cancer stem cells and formed miR208a/let-7 feedback loop via LIN28 and DICER1
Source: Oncotarget. 2015 Oct 8;6(32):32944–54. doi: 10.18632/oncotarget.5079 (PMC4741741; doi:10.18632/oncotarget.5079)
Supplement: Supplementary file 1 [file oncotarget-06-32944-s001.pdf]

SUPPLEMENTARY FIGURES

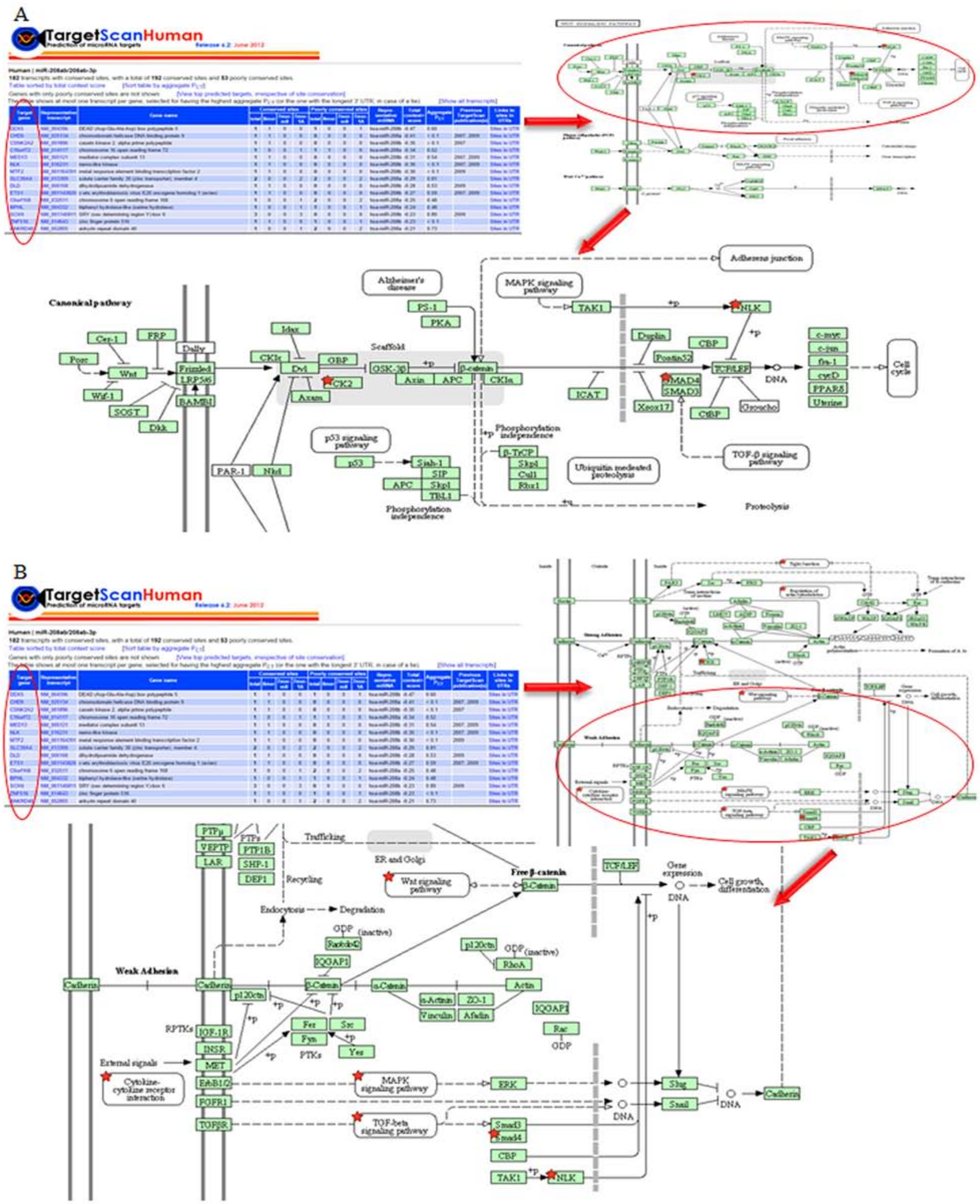

Supplementary Figure S1: Target predication of miR-208a downstream genes. Predication of downstream genes of miR-208a in website of "TargetScan Human" (http://www.TargetScan.com), and the predicated genes were pasted to David data base (http://david.abcc.ncifcrf.gov/summary.jsp). miR-208a may affect the Wnt signaling **A.** EMT and TGF-β pathway **B.** and in both of which Wnt signaling pathway was highly predicated and involved.

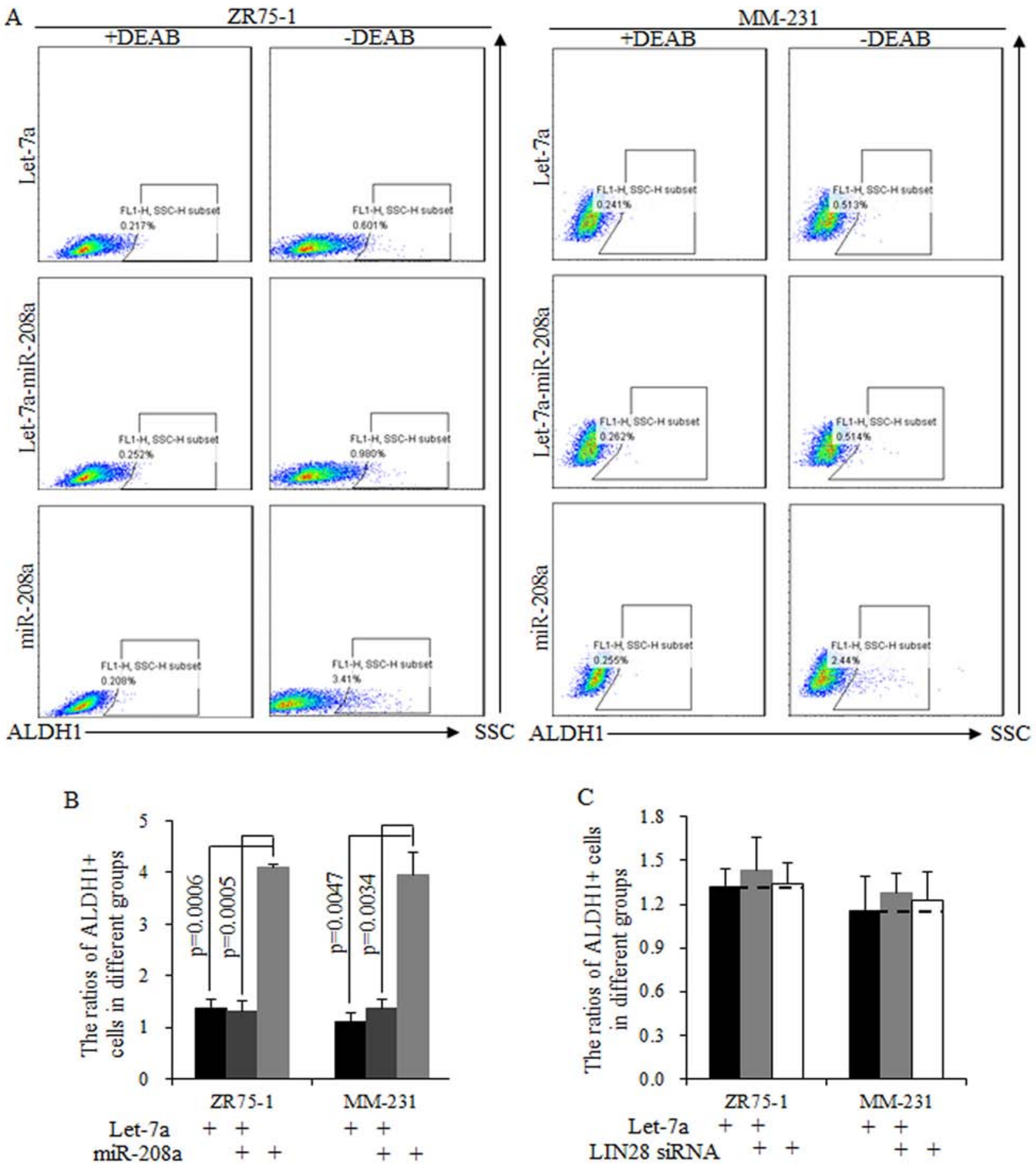

Supplementary Figure S2: Mir-208a promotes the stem cells renewal by repressing let-7a level via LIN28. A. Let-7a inhibits the self-renewal ability of breast cancer stem cells, and reversed the miR-208a induction of stem cell numbers, as were illustrated in **B. C.** LIN28 inhibition exhibits the same inhibitive functions on self-renewal, equals to let-7a.

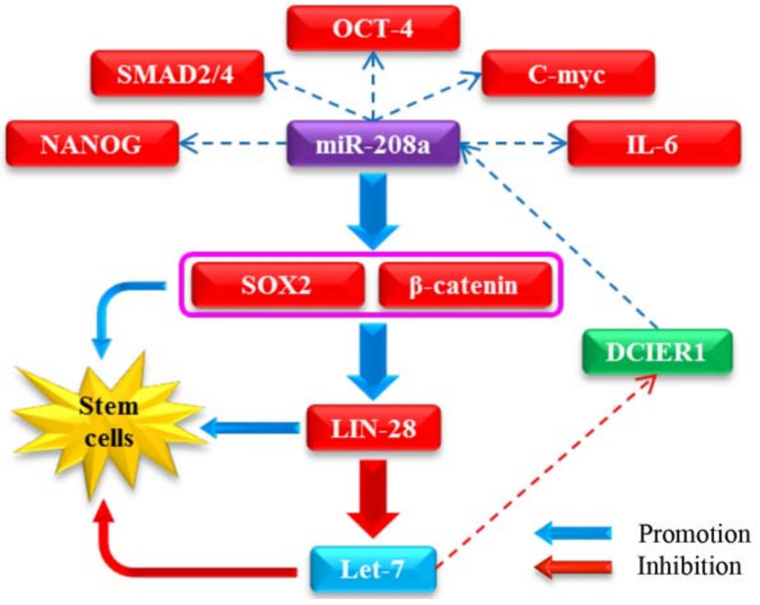

Supplementary Figure S3: The feedback loop between miR-208a and let-7a in breast cancer stem cells was constructed via SOX2/β-catenin, LIN28 and DICER1. The illustration diagram describes that miR-208a functions via regulations of SOX2 or β-catenin together, and miR-208a formed a negative feedback loop with let-7a through LIN28 and DICER1. MiR208a exhibits strong stimulation on LIN28 expression level, via promoting both SOX2 and β-catenin, which act as oncogenes in regulations of cancer stem cells; let-7a and miR-208a were connected to each other by LIN28 and DICER1.
